# Vitamin D and Sarcopenia: Potential of Vitamin D Supplementation in Sarcopenia Prevention and Treatment

**DOI:** 10.3390/nu12103189

**Published:** 2020-10-19

**Authors:** Ran Uchitomi, Mamoru Oyabu, Yasutomi Kamei

**Affiliations:** Graduate School of Life and Environmental Sciences, Kyoto Prefectural University, Kyoto 606-8522, Japan; s820731001@kpu.ac.jp (R.U.); s820631011@kpu.ac.jp (M.O.)

**Keywords:** vitamin D, sarcopenia, atrophy, skeletal muscle, gene regulation, nuclear receptor

## Abstract

Skeletal muscle, the largest organ in the human body, accounting for approximately 40% of body weight, plays important roles in exercise and energy expenditure. In the elderly, there is often a progressive decline in skeletal muscle mass and function, a condition known as sarcopenia, which can lead to bedridden conditions, wheelchair confinement as well as reducing the quality of life (QOL). In developed countries with aging populations, the prevention and management of sarcopenia are important for the improvement of health and life expectancy in these populations. Recently, vitamin D, a fat-soluble vitamin, has been attracting attention due to its importance in sarcopenia. This review will focus on the effects of vitamin D deficiency and supplementation on sarcopenia.

## 1. Sarcopenia

The term “sarcopenia” was first proposed by Rosenberg in 1989 and is derived from the Greek “sarx”, meaning flesh and “penia”, meaning loss [1,2]. Originally, sarcopenia referred only to the loss of skeletal muscle mass with aging. In 2010, the European Working Group on Sarcopenia in Older People (EWGSOP) defined sarcopenia as a syndrome characterized by the progressive and generalized loss of skeletal muscle mass and strength with a risk of adverse outcomes such as physical disability, poor quality of life (QOL), and death [3]. Another definition by the International Working Group on Sarcopenia described sarcopenia as an age-associated loss of skeletal muscle mass and function [4].

Sarcopenia has been associated with the propensity of elderly people to fall. It has been reported that sarcopenic individuals aged 80 years or older were over three times more likely to fall during a two-year observation period compared with non-sarcopenic individuals [5]. Furthermore, evidence from nine prospective cohort studies has shown that people with a high skeletal muscle mass and a fast walking speed have greater longevity as they are less likely to suffer from sarcopenia [6].

### 1.1. Etiology of Sarcopenia

Sarcopenia is classified as either primary or secondary based on etiological factors (Table 1). Sarcopenia is considered “primary” when no other specific cause other than aging is evident, while it is considered “secondary” when causal factors besides aging are apparent. Sarcopenia can occur secondary to a systemic disease, especially inflammatory diseases, e.g., malignancy or organ failure, and endocrine diseases such as diabetes mellitus. Physical inactivity also contributes to the development of sarcopenia. Moreover, sarcopenia can develop as a result of undernutrition or malabsorption.

### 1.2. Diagnosis of Sarcopenia

The most widely accepted method of diagnosing sarcopenia is from the EWGSOP, which considers the reduction in muscle mass and function (muscle strength or physical ability) as the standard criteria [3]. This has been further developed by a second convening of EWGSOP (EWGSOP2) to emphasize low muscle strength as the primary indicator of sarcopenia [7]. When low muscle strength is detected, it is increasingly likely that sarcopenia is present. The diagnosis is confirmed when low muscle strength is accompanied by low muscle quantity or quality. If physical performance is also poor, then the sarcopenia is considered severe.

Appendicular muscle mass is most commonly assessed using dual-energy X-ray absorptiometry (DXA) and bioelectrical impedance analysis (BIA). Walking speed (<0.8 m/s) and the 400 m walking test (>6 min) are recommended for the evaluation of physical performance [7]. The Asian Working Group for Sarcopenia (AWGS) has revised cutoff values for some diagnostic criteria: low muscle strength is defined as handgrip strength of <28 kg and <18 kg for men and women, respectively, reduced physical performance in the 6 m walking test is a speed of <1.0 m/s, and a time of ≥12 s in the chair stand test for five rises [8].

### 1.3. Prevalence of Sarcopenia

The prevalence of sarcopenia in 1882 elderly Japanese individuals, aged 65 to 89 years, was observed to be 21.8% and 22.1% for men and women, respectively, with diagnosis based on the EWGSOP criteria [9]. Other research with 243 elderly Thai participants indicated a sarcopenia prevalence of 30.5% (33.9% for men and 29.3% for women) [10]. Few studies have reported the prevalence of sarcopenia based on the EWGSOP2 criteria. EWGSOP2-defined sarcopenia prevalence was lower than that defined using EWGSOP-1 criteria due to the difference in diagnostic factors to detect sarcopenia. Reiss et al. reported a sarcopenia prevalence of 18.1% using the EWGSOP2 criteria versus a 27.7% prevalence with the EWGSOP criteria in 144 geriatric patients [11]. Similarly, in a study with 501 subjects, Locquet et al. observed a prevalence of sarcopenia of 7.4% and 13.6% for EWGSOP2 and EWGSOP, respectively [12].

On the other hand, the revisions made to the AWGS criteria appear to have the opposite effect on prevalence. In a study with 2123 elderly participants, aged 70–84 years, the prevalence of sarcopenia in men and women was 21.3% and 13.8%, respectively, with the revised AWGS criteria. The older AWGS criteria yielded a prevalence of 10.3% for men and 8.1% for women. In this case, revising the AWGS criteria seems to have relaxed the conditions under which sarcopenia is diagnosed [13]. The prevalence of sarcopenia is heavily dependent on the criteria that are used to diagnose it and since no criteria are used universally, the prevalence may vary widely.

## 2. Vitamin D

Vitamin D is a fat-soluble vitamin that can act as a hormone through a nuclear receptor. Vitamin D was discovered in cod liver oil as the anti-rickets factor in the 1930s. Since then, research on the metabolism of vitamin D has been conducted in a variety of species, and the metabolic pathways have been elucidated [14]. The most important function of vitamin D is in the regulation of Ca^2+^ concentration in the circulating blood, whose deficiency leads to diseases such as rickets in children and osteomalacia in adults [14]. It has recently been proven that deficiency or insufficiency in vitamin D is positively correlated with the risk of several diseases including sarcopenia, cardiovascular diseases, obesity, and cancer. Indeed, Remelli et al. reviewed the biological, clinical and epidemiological evidence supporting the association between vitamin D and an increased risk of sarcopenia in older people [15]. Consequently, attention has been drawn to the other aspects of vitamin D metabolism other than Ca^2+^ homeostasis. Vitamin D has significant effects on several other tissues [16], and the following sections will review its effects on skeletal muscle, including recent progress, and consequences on sarcopenia.

### 2.1. Synthesis and Metabolism of Vitamin D

**Figure 1:**
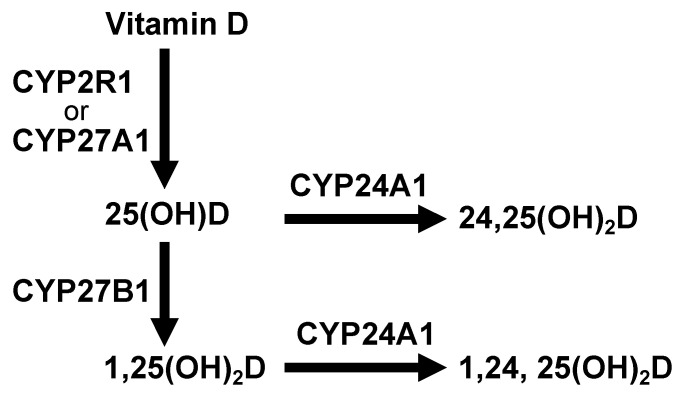
Vitamin D metabolic pathway by cytochrome P450 oxidases (CYPs). Vitamin D is hydroxylated at the C25 site by CYP2R1 or CYP27A1 in the liver leading to the production of 25-hydroxyvitamin D [25(OH)D]. The 25(OH)D is hydroxylated at the C1α site by CYP27B1 in the kidney, producing 1α,25-dihydroxyvitamin D [1,25(OH)_2_D]. Both 25(OH)D and 1,25(OH)_2_D are metabolized by CYP24A1, inactivated and in part excreted into the feces as bile or in urine.

Vitamin D is primarily produced in the skin when it has been exposed to ultraviolet (UV) rays. A product of cholesterol synthesis, 7-dehydrocholesterol, is converted into previtamin D by irradiation with ultraviolet light B (UVB) of wavelengths from 290 to 320 nm. The previtamin D is in turn non-enzymatically converted to vitamin D via double bond transfer which occurs at body temperature. However, it is required to ingest some dietary vitamin D to compensate for the shortage caused by insufficient de novo synthesis of vitamin D in the skin. Dietary vitamin D is divided into two groups: vitamin D_3_ (cholecalciferol), present in fish and egg yolk, and vitamin D_2_ (ergocalciferol) which is found in mushrooms. Approximately 80% of vitamin D is synthesized in the skin upon UVB exposure and the remainder is derived from the diet. These values may differ depending on factors such as ethnicity, duration of exposure to sunlight, and season [17,18]. Vitamin D is absorbed in the small intestine, then incorporated into chylomicrons that are transported via lymphatic vessels into the veins for distribution throughout the body. Serum vitamin D is conjugated to a vitamin D binding protein and transported to the liver where vitamin D is metabolized. Metabolism of vitamin D is mediated by cytochrome P450 oxidases (CYPs) [19] (Figure 1). Vitamin D is hydroxylated at the C25 site by CYP2R1 or CYP27A1 in the liver leading to the production of 25-hydroxyvitamin D [25(OH)D]. Serum 25(OH)D levels are used to determine vitamin D sufficiency. The 25(OH)D is hydroxylated at the C1α site by CYP27B1 in the kidney, producing 1α,25-dihydroxyvitamin D [1,25(OH)_2_D]. The 1,25(OH)_2_D then binds to a vitamin D receptor (VDR), a nuclear, ligand-dependent transcription factor, eliciting several physiological responses through the regulation of multiple VDR target gene expressions. Both 25(OH)D and 1,25(OH)_2_D are metabolized by CYP24A1, inactivated and in part excreted into the feces as bile or in urine [20] (Figure 1).

### 2.2. Vitamin D Deficiency and Hypervitaminosis D

Vitamin D deficiency reduces calcium and phosphorus absorption from the intestinal tract. This results in hypocalcemia and hypophosphatemia which can cause rickets in children and osteomalacia in adults [20]. The primary cause of vitamin D deficiency is reduced vitamin D synthesis in the skin which can be caused by: inadequate UV exposure, excessive use of sunscreen, and limited outdoor activity. Deficiency is also associated with decreased ingestion of dietary vitamin D, aging, and hepatic or renal disorders. The nutritional status of vitamin D is evaluated by measuring the serum 25(OH)D concentration. A concentration of 30 ng/mL is indicative of vitamin D insufficiency and that of 20 ng/mL and less reflects deficiency [21].

Hypervitaminosis D, excess vitamin D, is responsible for hypercalcemia, renal dysfunction, and nephrocalcinosis. However, vitamin D is relatively safe as hypervitaminosis D is caused by the substantial consumption of vitamin D over an extended period (a few months). Furthermore, elevated 1,25(OH)_2_D levels due to increased intake of vitamin D inhibit the activity of the renal enzyme CYP27B1 and stimulate CYP24A1 activity, leading to reduced serum 1,25(OH)_2_D levels [22].

### 2.3. Regulation of Gene Expression by the Vitamin D/VDR

**Figure 2:**
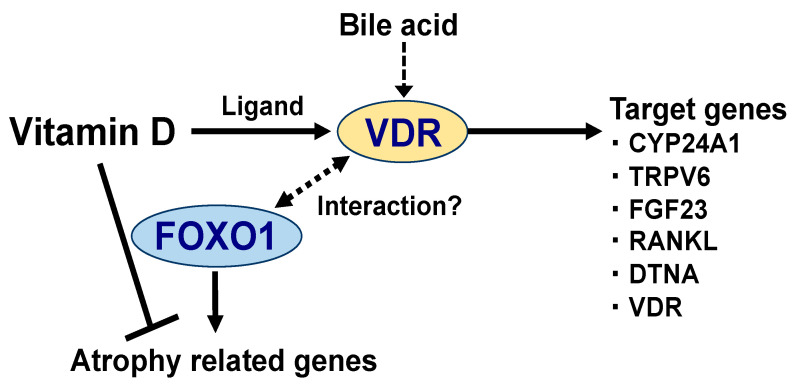
Vitamin D regulates the expression of the target genes by binding to the vitamin D receptor (VDR). The primary target genes for VDR [28,29,30,31,32,33] are shown in Figure 2. CYP24A1, cytochrome P450 family 24 subfamily A member 1; TRPV6, transient receptor potential cation channel subfamily V member 6; FGF23, fibroblast growth factor 23; RANKL, receptor activator of nuclear factor-kappaB-ligand; DTNA, dystrobrevin alpha; and VDR. In skeletal muscle, vitamin D has been shown to suppress the expression of muscle atrophy-related genes (atrogin-1 and cathepsin L). Possibly, this is achieved by the ability of vitamin D to repress the transcriptional activity of the forkhead box protein O1 (FOXO1), which activates the genes involved in protein degradation (discussed in later section). FOXO1 has been reported to physically interact with multiple nuclear receptors (possibly with VDR) [37,38], which may be responsible for the suppression of atrophy-related gene expression. However, further investigations are required to shed light on the relationship between vitamin D and FOXO1.

The VDR is a nuclear receptor, ligand-dependent transcription factor [23], which forms complexes with co-factor proteins [24,25]; these complexes regulate gene expression in numerous physiological processes. Once the 1,25(OH)_2_D binds to the VDR as a ligand, VDR interacts with its heterodimer partner, retinoid X receptor, and subsequently binds to the vitamin D response element (VDRE) located on the target genes [26,27]. Activated VDR induces the gene expression of CYP24A1 (Figure 2), which inactivates 1,25(OH)_2_D by hydroxylation at C24, as a negative feedback machinery [28]. Transient receptor potential vanilloid—subfamily V, member 6 (TRPV6), a calcium channel in the small intestinal mucous membrane—is also a target gene of VDR [29]. In addition, there are more VDR-target genes, such as fibroblast growth factor-23 (FGF23) and receptor activator of NF-kappaB ligand (RANKL), involved in calcium and phosphate homeostasis [30,31]. Recently, dystrobrevin alpha (DTNA), a member of the dystrophin-associated protein complex (DPAC), was also identified as the VDR-target gene in skeletal muscle cells [32]. Moreover, 1,25(OH)_2_D increases the expression of the VDR gene itself [33] (Figure 2). Meanwhile, the activation of VDR has been reported to be caused by bile acids, as an endogenous ligand [34,35] (Figure 2). Vitamin D is also known to act independently of VDR. Recently, Asano et al. demonstrated that 25(OH)D induces the degradation of sterol regulatory element-binding protein (SREBP) cleavage-activating protein (SCAP) without VDR, consequently suppressing SREBP-2, a transcription factor that induces cholesterol synthesis [36].

## 3. Vitamin D and Sarcopenia

### 3.1. Vitamin D Deficiency and Sarcopenia

A positive correlation has been shown to exist between serum 25(OH)D concentration and muscle function. Serum 25(OH)D concentrations <30 ng/mL (75 nM) and <20 ng/mL (50 nM) are indicative of vitamin D insufficiency and deficiency, respectively [21]. In a study by Okuno et al., vitamin D insufficiency was reported in 89% and deficiency in 28% of a sample of 80 elderly Japanese women over the age of 65 years [39]. Of those whose vitamin D levels were insufficient or deficient, 56.3% experienced falls during a three-month observation period. In another report, a meta-analysis of five randomized controlled trials investigating the effects of vitamin D supplementation (20 µg/day, 800 IU/day) on falls and bone fractures in the elderly revealed that vitamin D supplementation lowered the risk of falling by 22% compared with calcium alone or a placebo. Furthermore, vitamin D supplementation at 20 µg/day resulted in a significantly lower incidence of bone fracturing compared with a supplementation rate of 10 µg/day (400 IU/day) [40]. It has been concluded that elderly individuals with low serum 25(OH)D concentrations are susceptible to sarcopenia [41]. A decline in serum 25(OH)D concentration with advanced age results in reduced bone density, leading to a higher risk of falling and bone fractures. It has been observed that the expression of CYP24A1, an enzyme that inactivates 1,25(OH)_2_D, increases with age in the rat kidney [28]. MacLaughlin and Holick concluded that reduced outdoor activity with aging results in a decline in the ability to synthesize vitamin D and a two-fold decrease in previtamin D production by the skin [42]. These factors culminate in low serum 25(OH)D concentrations in elderly individuals. Another consequence of aging is the reduced ability to synthesize 1,25(OH)_2_D in the kidneys [43].

Sarcopenic patients often become obese (sarcopenic obesity) as a result of the negative correlation that exists between serum 25(OH)D concentration and body fat mass. Vitamin D deficiency is frequently observed in obese people [44]. It has been shown that vitamin D inhibits the differentiation of 3T3-L1 preadipocytes to mature adipocytes [33,45]. It follows that low serum concentrations of 25(OH)D would mean that there is a reduced inhibitory effect on the differentiation of preadipocytes, hence obesity becomes probable.

### 3.2. Vitamin D and Muscle Strength

Studies on vitamin D supplementation have shown an increase in muscle strength due to supplementation. A systematic review of twenty-nine studies investigating the implications of vitamin D supplementation on muscle strength revealed that vitamin D supplementation significantly increased muscle strength—more so in individuals with serum 25(OH)D concentrations <30 ng/mL versus those with >30 ng/mL. This implies that vitamin D supplementation is more effective in cases were serum 25(OH)D concentrations are low, as is the case with elderly individuals [46]. A separate study also showed that muscle nuclear VDR was increased by 30% and augmented muscle fiber size by 10% in elderly females (mean age of 78 years) taking vitamin D orally at a rate of 100 µg/day (4000 IU/day) for 4 months [47]. On the contrary, a meta-analysis of seven controlled trials with vitamin D supplementation showed an improvement in upper and lower limb muscle strength in healthy 18–40-year-old participants. This shows that the benefits of vitamin D supplementation are not limited to the elderly and frail [48]. The administration of vitamin D may improve muscle strength and muscle mass and may be useful for the prevention and therapeutic intervention of sarcopenia. However, vitamin D supplementation does not always improve muscle function as seen in the meta-analysis of 16 randomized, controlled trials investigating the effects of vitamin D supplementation on muscle function in postmenopausal women. Vitamin D supplementation did not improve grip strength and back muscle strength, which are indicators of general muscle strength [49]. These differences in the effects of vitamin D supplementation may be due to several reasons such as the amount and type of vitamin D used, the duration of the intervention, and the state of vitamin D sufficiency in the subjects. Further research is needed in this area.

## 4. Mechanism of Action of Vitamin D on Skeletal Muscle

### 4.1. Expression of VDR in Skeletal Muscle

Vitamin D is metabolically converted into active 1,25(OH)_2_D by the metabolic enzyme CYP27B1 in the kidney, and subsequently binds to the nuclear receptor VDR to regulate the target gene expression. The expression of VDR and CYP27B1 was observed in neonatal and damaged skeletal muscle, and to a lesser extent in mature skeletal muscle tissue [50,51]. Systemic VDR knockout (KO) mice have been observed to have reduced muscle mass, muscle fiber size, and muscle strength (grip strength) compared with wild-type mice [52]. Another phenotypic study with VDR-KO mice showed muscle fiber size atrophy at three weeks of age and pronounced muscle atrophy at eight weeks. There was also delayed skeletal muscle maturation characterized by persistent immature neonatal-type myosin heavy chain expression up to 3 weeks of age in skeletal muscle of the VDR-KO mice [53]. Similarly, in skeletal muscle-specific VDR-KO mice, decreased voluntary activity in rotating cages, declined muscle function (examined as lower grip strength), and reduced muscle mass were all recorded. Additionally, the expression of sarcoendoplasmic reticulum calcium transport ATPase (SERCA) and Calbindin, genes involved in the regulation of intracellular calcium concentration, decreased in skeletal muscle of skeletal muscle-specific VDR-KO mice [54]. These reports suggest that vitamin D improves muscle function and muscle mass through the mediation of VDR, although the detailed mechanisms are still unclear.

### 4.2. Vitamin D Effects on the Expression of Atrophy-Related Genes

Vitamin D can suppress the activity of atrophy-related transcription factors. We have been investigating the mechanisms underlying the regulation of skeletal muscle metabolism by FOXO1. Based on the fact that energy deprivation increases FOXO1 gene expression in skeletal muscle, genetically modified mice with an overexpression of FOXO1 specifically in skeletal muscle have been generated and used to show that FOXO1 causes muscle atrophy [55]. The increased expression of FOXO1 is commonly observed during muscle atrophy under multiple pathophysiological conditions such as malnutrition, inactivity, and cancer [56,57]. FOXO1 and its analog FOXO3a are known to induce muscle atrophy by mechanisms that include enhanced protein degradation through the ubiquitin-proteasome system and autophagy induction [55,58]. A reporter assay system was prepared with the intention of measuring the transcriptional activity of FOXO1. After screening several compounds derived from various foods and plants, it was found that 1,25(OH)_2_D suppressed the transcriptional activity of FOXO1 [59]. Moreover, 1,25(OH)_2_D suppressed the increased expression of atrogin-1 and cathepsin L, target genes for FOXO1, which induces muscle atrophy in C2C12 myoblasts [59] (Figure 2). Interestingly, Yang et al. recently reported that physical inactivity and low serum vitamin D can synergistically promote sarcopenia in the elderly [60]. Under inactive conditions, vitamin D deficiency accelerated the loss of muscle mass, muscle cross-sectional area, and grip strength, and conversely increased protein expression of FOXO3a and its target genes (Atrogin-1 and MuRF1). In older adults, serum 25 (OH) D_3_ and physical activity showed interactive effects on physical performance (timed up and go test) and muscle strength (grip strength) [60].

### 4.3. Vitamin D Effects on Protein Synthesis and Skeletal Muscle Hypertrophy

Recent evidence indicated that vitamin D can also stimulate protein synthesis via mammalian target of rapamycin complex 1 (mTORC1) signaling and induce skeletal muscle hypertrophy. Bass et al. demonstrated that overexpression of VDR in rats induced muscle hypertrophy, which was characterized by the increased muscle cross-sectional area, and that it enhanced anabolic signaling and translational efficacy, resulting in increased phosphorylated mTOR (p-mTOR) and downstream targets (p-4E-BP and p-p70S6K) [61]. Conversely, vitamin D deficiency in rats inhibited mTORC1 signaling and contributed to decreased protein synthesis in skeletal muscle [62]. These reports suggest that VDR in skeletal muscle plays important roles in muscle hypertrophy.

### 4.4. Effects of Vitamin D on Skeletal Muscle Mitochondria

Mitochondrial dysfunction results in mild but chronic inflammation due to increased production of reactive oxygen species, leading to qualitative/quantitative deterioration of skeletal muscle, which is thought to be one of the major causes of sarcopenia onset [63]. Reports suggest that 1,25(OH)_2_D supplementation improves the function of mitochondrial oxidative phosphorylation in the skeletal muscle in vitamin D deficient humans [64]. Furthermore, the introduction of 1,25(OH)_2_D to human skeletal muscle cells enhances the oxygen consumption rate of mitochondria and activates pyruvate dehydrogenase [65]. The regulation of mitochondrial respiration via 1,25(OH)_2_D is dependent on the VDR because silencing of the VDR in skeletal muscle cells reduces mitochondrial oxygen consumption rate and adenosine triphosphate (ATP) production derived from oxidative phosphorylation [66]. In addition, 1,25(OH)_2_D also ameliorates the palmitic acid (PA)-induced mitochondrial dysfunction and triglyceride (TG) accumulation in skeletal muscle cells [67]. In elderly individuals aged 60 to 80, vitamin D has been reported to reduce intramyocellular lipid accumulation in combination with treadmill aerobic training [68]. Hence, 1,25(OH)_2_D may have beneficial effects on skeletal muscle by regulating mitochondrial function.

## 5. Guidelines of Vitamin D Intake and Actual Intake

The Dietary Reference Intake (2020) in Japan determines the Adequate Intake (AI) for vitamin D which was recently reviewed upwards from 5.5 µg/day (220 IU) to 8.5 µg/day (340 IU) (Table 2) [69]. However, this value is still much lower than in other countries. Previously, the Dietary Reference Intake of the United States and Canada specified the AI for vitamin D, but changed to the Estimated Average Requirement and Recommended Dietary Allowance in 2011 [70]. The recommended quantities are: 15 µg/day (600 IU) for people under 70 years old; 20 µg/day (800 IU) for those 71 years and older. According to the International Osteoporosis Foundation, dietary vitamin D intake from 20 to 25 µg/day (800 to 1000 IU/day) is required to prevent both falls and bone fractures in elderly women (Table 2) [71].

## 6. Vitamin D Fortified Foods

Dairy products make up the bulk of the foods that are fortified with vitamin D. Programs to develop vitamin D fortified foods such as milk, margarine, and yogurt have been introduced in several countries, including the United States, Canada, and Finland [18]. A recent study reported that garden pea protein-based, small vitamin D nanoemulsion (233 nm) increased the efficiency of vitamin D transport into Caco-2 cells by up to 5.3 times that of a free vitamin D suspension [72]. Similarly, Almajwal et al. found that Wistar rats fed a vitamin D deficient diet (vitamin D < 50 IU/kg (25 µg/kg)) for 6 weeks were soon restored to normal serum 25(OH)D levels by oral administration of garden pea protein-based vitamin D fortified nanoemulsion (containing 81 µg of vitamin D) given every other day for one week (three times in total). In contrast, there was no improvement in serum 25(OH)D in the group given canola oil supplemented with the same amount of vitamin D [73]. In the future, studies on the development of foods fortified with vitamin D by way of nanoemulsion technology, as well as functional foods containing vitamin D, may significantly increase the bioavailability of vitamin D in the elderly, ultimately contributing to improved vitamin D deficiency interventions.

## 7. Closing Remarks

In developed countries with large proportions of elderly citizens, the prevention of sarcopenia and the management of its progression are important issues in terms of reducing health care costs and improvement of QOL. Evidence is given from several studies on the efficacy of vitamin D as an intervention in cases of sarcopenia in the elderly. However, whether supplementation with vitamin D in sarcopenia patients has beneficial effects such as suppression of muscle atrophy and increased muscle strength is controversial, in part because of the complicated mechanisms underlying the action of vitamin D on muscle tissue. Further studies on vitamin D and sarcopenia will be useful in shedding more light on the matter.

## Figures and Tables

**Table 1:** Etiology of primary and secondary sarcopenia.

Categories	Causes
**Primary sarcopenia**	
Aging	Age-related muscle loss
**Secondary sarcopenia**	
Disease	Inflammatory conditions (e.g., malignancy or organ failure) Endocrine disease (e.g., diabetes mellitus)
Inactivity	Sedentary lifestyle (e.g., bedridden, dependent on a wheelchair or disease-related limited mobility)
Malnutrition	Undernutrition or malabsorption Medication-related anorexia

**Table 2:** Comparison of vitamin D requirements.

**Dietary Reference Intake for Japanese**	
2005	5.0 µg/day (200 IU)
2015	5.5 µg/day (220 IU)
2020	8.5 µg/day (340 IU)
**Dietary Reference Intake for the U.S. and Canada (2011)**	
9–70 years	15 µg/day (600 IU)
>70 years	20 µg/day (800 IU)
**International Osteoporosis Foundation (IOF) Position Statement**	
Fracture prevention	20 µg/day (800 IU)
Fall prevention	25 µg/day (1000 IU)
